# Feasibility and potential cognitive impact of a cognitive-motor dual-task training program using a custom exergame in older adults: A pilot study

**DOI:** 10.3389/fnagi.2023.1046676

**Published:** 2023-02-02

**Authors:** Matthieu Gallou-Guyot, Stephane Mandigout, Romain Marie, Louise Robin, Jean-Christophe Daviet, Anaick Perrochon

**Affiliations:** ^1^Laboratoire HAVAE (UR20217), Université de Limoges, Limoges, France; ^2^Institut d’Ingénierie Informatique de Limoges, Limoges, France; ^3^3iL Ingénieurs, Limoges, France; ^4^Center for Interdisciplinary Research in Rehabilitation and Social Integration (CIRRIS), Québec City, QC, Canada; ^5^Pôle Neurosciences Tête et Cou, Service de Médecine Physique et de Réadaptation, Hôpital Jean Rebeyrol, CHU Limoges, Limoges, France

**Keywords:** exergame, training, older adults, cognitive motor dual task, feasibility

## Abstract

**Introduction:**

Dual-task training may be relevant and efficient in the context of active aging. An issue in training programs lies in enhancing the adherence of participants. This can potentially be improved using games as support. We designed and developed a custom interactive exergame in this way. The objective of this pilot study was to explore the potential use of this exergame and the feasibility of our intervention, including the level of safety and adherence. The result’s trends on cognitive and motor capacities, as well as on the level of motivation for physical activity, fear of falling, and quality of life of participants, were also explored.

**Methods:**

Older adults aged 65 years or older were recruited and realized 30 min of supervised training in groups of 4, 2–3 times a week for 12 weeks. Exercises consisted of incorporated cognitive and motor dual tasks, with an increased difficulty over the weeks. Our program’s safety, engagement, attendance, and completion levels were evaluated. Participants’ postural control in single-task and dual-task conditions, as well as their performances in mental inhibition, flexibility, working memory, mobility, and postural control, and their levels of motivation for physical activity, fear of falling, and quality of life were also assessed. We realized a per protocol statistical analysis with a *p*-value set at 0.05.

**Results:**

Thirty-nine participants (aged 84.6 ± 8.5 years) were recruited. No adverse events, and 89% adherence, 88% attendance, and 87% completion rates were observed. A potentially significant effect of our exergame on working memory in single-task conditions and on the cognitive aspect of dual-task conditions was also observed. We observed no differences in other parameters.

**Discussion:**

Our exergame seemed feasible and safe and was enjoyed by participants, mainly due to the gamification of our training program. Moreover, our exergame may be efficient for cognitive training in older adults, as well as for the maintenance of motor functions, motivation for physical activity, fear of falling, and quality of life levels. This constitutes the first step for our solution with interesting results that need to be further studied.

## 1. Introduction

Aging is associated with cognitive (Park, 2002; Verhaeghen and Cerella, 2002; Zhou et al., 2011) and motor (Greenlund and Nair, 2003) capacities declines. Unsurprisingly, older adults also present a decrease in cognitive-motor dual-task (DT) capacities (Montero-Odasso et al., 2012; Yogev-Seligmann et al., 2012), reflecting an increase in CMIs (Yogev-Seligmann et al., 2008). Several cognitive and motor capacities affected during aging represent fall risk factors, such as executive functions, reaction time, processing speed (Segev-Jacubovski et al., 2011) or postural control, motor coordination, muscle strength, walking, and mobility (Judge et al., 1996; Al-Aama, 2011). Moreover, CMI in older adults is also associated with an increased risk of falls (Al-Aama, 2011; Segev-Jacubovski et al., 2011) and, therefore, a loss of autonomy (Anton et al., 2015; Tornero-Quiñones et al., 2020) or even a deterioration in the quality of life (Figueira et al., 2008; Anton et al., 2015). Facing the important frequency (Campbell et al., 1981) and high impact (World Health Organization, 2008) of falls in older adults, the maintenance of these cognitive, motor, and DT capacities is essential from an active aging perspective.

In this context, we know that DT trainings are relevant and efficient for the improvement of cognitive (Wollesen et al., 2020; Gavelin et al., 2021), motor (Varela-Vásquez et al., 2020; Gavelin et al., 2021), and DT capacities (Bherer et al., 2020) in normal aging. DT trainings are defined as physical (Fissler et al., 2013) or motor (Gavelin et al., 2021) training programs, including an added or incorporated cognitive task (Herold et al., 2018) realized simultaneously or sequentially (Tait et al., 2017). DT trainings are, therefore, varied both in form and content: they can either be realized alone or in groups, supervised or not, using tools and apparatus as support or not, with highly variable intensity, length, frequency, and duration modalities (Gallou-Guyot et al., 2020a). A recent study proposed a new categorization of DT training based on the key characteristics that are stimuli, settings, targets, markers, and outcomes (Torre and Temprado, 2022a). Beyond this great variability making intercomparison difficult, a critical issue with physical or motor training programs in older adults is to obtain their adherence (Jancey et al., 2007; Hawley, 2009; Nyman and Victor, 2012), gathering their engagement, attendance, compliance, and completion (Kolt et al., 2007). Participants’ adherence is a key point to focus on, as training’s efficiency depends on their effective realization (Robison and Rogers, 1994).

Engagement in training programs can be improved using games as support. Indeed, a strong argument for gamified physical interventions would be their attractiveness, i.e., their ability to arouse interest in the target audience as they seem appealing and enjoyed (Koivisto and Hamari, 2019). Older people’s compliance and completion rates to programs using exergames are, thus, higher than that of conventional interventions (Choi et al., 2017). This would be due to the continuous and instant feedback (Lyons, 2014) as well as the fun aspect of exergames (Mellecker et al., 2013), which increases people’s participation and completion rates by increasing their pleasure in participating (Mellecker et al., 2013; Lyons, 2014). This can be explained through the gamification theory, i.e., “the use of elements from games in a context other than the game” (Deterding et al., 2011). The physical activity practice’s gamification also seems to obtain beneficial effects on the engagement and maintenance of physical activity (Koivisto and Hamari, 2019). The means of the action of gamification are to make the player progress by earning points, to integrate a feedback mechanism (or engagement loop), to encourage exchanges between players, and to allow the personalization of the service (Deterding et al., 2011).

Exergames are defined as active video games that require simultaneous cognitive and physical activities (Vázquez et al., 2018). Played on digital devices, they include a wide range of interfaces (Baranowski et al., 2008) and content (Kappen et al., 2019). Once more, facing this great variability, a recent study proposed a new categorization of exergames based on the same key characteristics (Torre and Temprado, 2022b). Although they allow delivering DT and have been considered a special form of DT training for a long, a recent study distinguished exergames from DT trainings (Temprado and Torre, 2022) based on their different effects. Indeed, exergames have been widely studied in older adults and are considered by many authors to be efficient for the improvement of cognitive functions (Soares et al., 2021; Temprado and Torre, 2022), but their effects on motor functions are more discussed (Pacheco et al., 2020; Chen et al., 2021; Ge et al., 2021). Moreover, the effects of exergames on DT functions are still less studied (Gallou-Guyot et al., 2020a). Additionally, the exergames used for training in older adults are mostly commercial solutions (Bonnechère et al., 2016), although the efficiency of training programs depends on their personalization to the participant’s characteristics and their objectives (Robert et al., 2014; Manera et al., 2017). For instance, recommendations for custom exergames used in older adults are almost never met with commercial solutions regarding the ease of use: slow animations, large fonts, simple menus, and rules (Chao et al., 2015).

The need to fit participants’ specificities and objectives, the missing information in the literature concerning the effect of exergames on DT functions in older adults, and their recent differentiation from DT trainings led us to design and develop a custom exergame (Gallou-Guyot et al., 2022a) that would meet the needs of older adults, targeting cognitive and motor risk factors for falls using the concept of CMI. Rehabilitation interventions are complex: interacting components, numerous and variable outcomes, and highly flexible intervention, for instance. Given this complexity, pilot and feasibility studies are needed to evaluate whether the solution proposed can be implemented (Kho and Thabane, 2020). The objective of this pilot study was to explore the potential use of our exergame and the feasibility of the intervention through the level of safety, engagement, attendance, and completion. We explored the result’s trends on the cognitive, motor, and DT capacities in older adults, as well as on the level of motivation for physical activity, fear of falling, and quality of life of the participants.

## 2. Materials and methods

This study was constructed in accordance with the CONSORT extension to pilot and feasibility trials (Eldridge et al., 2016). The method of this prospective multicentric pilot study has been published as a trial (Gallou-Guyot et al., 2022a), in which we detailed all the participant eligibility and recruitment, the study design, intervention, outcomes, and statistical analysis.

### 2.1. Participants

Older adults aged 65 years or above were recruited, all residents of three different Limoges’ community dwellings. The principal investigator introduced the study to potential participants. Volunteers were received individually for an eligibility study. If they met the inclusion criteria and gave their written consent, they were included and received an information notice. They had to present normal or corrected vision and hearing, with no diagnosed pathology affecting walking and balance; double cane and walker technical assistance were not accepted. The sample size calculation was based on a previous study by Fraser et al. (2017), assessing postural control during a concurrent cognitive task. We hypothesized that the program would improve the center of pressure oscillation speed (mm/s) when performing a Stroop test by 30%. We performed an estimation with 0.80 power and 0.05 alpha risk. The resulting required sample size was 32 subjects. We added 20% to ensure the statistical power of the study, taking into account dropouts. As a result, the total sample was 39 participants.

This project received authorization from the ethics and individual protection committee Sud-Est 2 (specific reference number: 2020-A02805-34). This project was registered at ClinicalTrials.gov (NCT04803799).

### 2.2. Experimental design

All participants realized 30 min of training, 2–3 times a week for 12 weeks (odd and even weeks) for a total of 30 sessions. Training sessions were systematically realized in collaborative groups of 4 (10 groups in total), supervised by an animator, and dispensed within community dwellings. Training sessions consisted of DT exercises realized using our custom exergame as support. Exercises were of progressive difficulty and consisted of incorporated cognitive and motor tasks realized simultaneously (Herold et al., 2018), with a duration of 3 min per exercise. An incremental program was initially designed, and the difficulty was adapted during the sessions (speed, number of repetitions, task’s complexity, use of gymnastic equipment, etc.). Cognitive and motor tasks involved as falls risk factors in older adults were targeted: executive functions and processing speed (Tornero-Quiñones et al., 2020) on the one hand, and muscular strength, balance, stepping, and gait (Al-Aama, 2011) on the other. For instance, arrows were displayed successively on the projected scene, and participants had to reproduce them on a pad with one foot, two feet, doing a squat, a lunge, etc. In this example, the additional cognitive tasks were to not reproduce an arrow, to invert them, or to render them with a delay. Our training was designed following the most recent recommendations on DT training and exergaming (Gallou-Guyot et al., 2020a), prescription of physical activity (American College of Sports Medicine and Pescatello, 2014), and fall prevention (Sherrington et al., 2017) in older adults.

**FIGURE 1:**
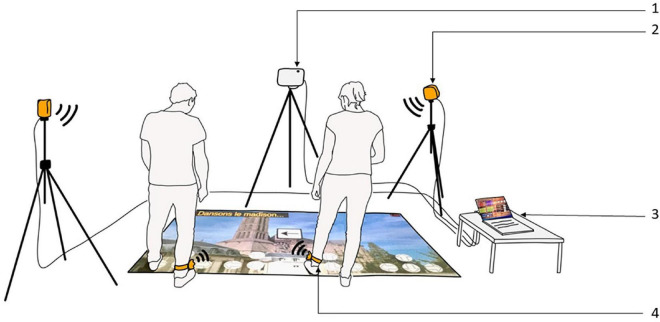
The exergame. (1) Video projector, (2) HTC vive infrared camera, (3) computer, and (4) HTC vive infrared tracker. In this illustration, only two participants are represented, the objective being to maximize visibility.

Our exergame consisted of a projection of the city of Limoges onto the ground as a game board. The aim of the game was for players to explore the main places in the city, which hosted mini-games involving DT collective exercises lasting an average of 3 min. The players had to collectively obtain the highest possible score. The scores were assigned by the animator for each mini-game as follows: a player’s presence counted as 1, his/her participation as 2, and his/her satisfactory achievement as 3. The space needed for the game setting was 5 m × 5 m. This allowed the projection of the board game on the ground (3 m × 4 m) while keeping space for participant displacement and chair placement for any needed pause. HTC Vive infrared trackers and cameras were used to trigger the scenes’ changes between mini-games. We developed our own software for all the human–machine interaction using Unity (board game, transitions, mini-games, scoring, etc.) [refer to Figure 1 for an illustration of the exergame and refer to Gallou-Guyot et al. (2022a) for details on the training program, as well as on our exergame, its rules and use, different interfaces, performance charts, etc.].

### 2.3. Outcomes

#### 2.3.1. Feasibility

Intervention parameters include safety (number of serious adverse events have occurred during the intervention or directly related to it), engagement (number of eligible people that participated), attendance (number of proposed sessions realized), and completion (number of participants going to the end of the program) were assessed.

#### 2.3.2. Potential effects

The differences before (T1) and after (T2) 12 weeks of exergaming on several outcomes were evaluated. The approximate duration of the assessment was 2 h per participant.

The postural control under DT condition, measured through the center of pressure speed oscillation (mm/s) on a stabilometric platform (Win-Posturo, Médicapteurs^®^) during the performance of a concurrent Stroop test, was evaluated. Postural control in the dual-task condition is widely used in studies involving elderly persons (Dault and Frank, 2004; Westlake and Culham, 2007; Doumas et al., 2008; Heiden and Lajoie, 2010; Hiyamizu et al., 2012; Uemura et al., 2012; Melzer and Oddsson, 2013; Fraser et al., 2017; Bruce et al., 2019). During the evaluation, the participant was standing still on the platform, arms along the body. The participant was instructed to actively control his posture, standing as still as possible, and to perform a Stroop test during the 30-s evaluation. The Stroop test was presented on a poster (2 m × 1 m) pinned on the wall, 1.5 m away from the platform.

The cognitive and motor capacities of participants in the single-task condition were also evaluated, as well as their level of motivation for physical activity, fear of falling, and quality of life. The tests were as follows: (1) mental inhibition: during the Stroop test (Stroop, 1935), the participant must distinguish the name of the written color from the color of the ink used. Scoring included the time to complete the test (s) and the rate of errors. (2) Mental flexibility: during the TMT (Reitan, 1958), the participant must link a consecutive sequence of 25 targets in ascending order; initially, numbers (1, 2, 3, etc.) in part A and then alternate between numbers and letters (1, A, 2, B, 3, C, etc.) in part B. Scoring consisted in the difference of time to complete the two parts (B-A) (s). (3) Working memory: during the visual N-Back test (Kirchner, 1958), a continuous sequence of letters was presented to the participant. The task consisted of continuously indicating the letter previously displayed. The score corresponded to the rate of errors. (4) Mobility: during the TUG test (Podsiadlo and Richardson, 1991), the subject had to rise from his/her chair, walk 10 feet, turn around a mark, return to the chair, and sit down. We measured the time taken to complete the test (s). (5) Balance: during the BBS test (Berg et al., 1989), the subject was required to complete 14 balance tasks, ranging from getting up from a chair to standing on one leg. Scoring was done on a scale of 0–56 corresponding to the 14 items. Association between TUG sensitivity and BBS specificity is recommended in evaluation of the elderly (Park, 2018). (6) Quality of life was assessed with the EuroQol 5 dimensions 5 levels survey (EQ-5D-5L) (Janssen et al., 2013). Scoring was done on five scales of 1–5, and a list of 0–100. (7) Motivation for physical activity was assessed with the French motivation for physical activity for health scale (EMAPS) (Boiché et al., 2019). Scoring was done on six scales of 3–21. (8) Fear of falling was assessed with the FES-I (Yardley et al., 2005), which explores the participant’s concern about the possibility of falling while performing activities. Scoring was done on a scale of 16–64 corresponding to the 16 items.

### 2.4. Statistical analysis

A per-protocol statistical analysis using RStudio (RStudio, Inc.) was realized with a *p*-value set at 0.05. The normality of distributions was tested using Shapiro–Wilk tests. The training potential effects (T1–T2) were tested using the dependent Student’s *t*-test or Wilcoxon signed-rank test depending on the normality of distribution. The type I error rate was corrected using the Bonferroni–Holm method (Holm, 1979). The effect size was calculated using Cohen’s *d* (Cohen, 1988; Sawilowsky, 2009).

## 3. Results

### 3.1. Population description

Among 175 residents, 44 older adults were eligible to engage in our study, of whom 39 participated, representing an 89% engagement rate. Our sample consisted of 10 men and 29 women with an average age of 84.6 ± 8.5 years, a height of 161.1 ± 8.1 cm, a weight of 66.5 ± 12.0 kg, and a body mass index of 25.6 ± 4.3. They were retired for 19.5 ± 2.1 years. Twenty-one participants had a single cane, while others did not have any technical help. Regarding the maximum levels of education, 28 participants had reached the primary level, 9 a secondary level, and 2 an equivalent of bachelor’s degree.

### 3.2. Feasibility

**FIGURE 2:**
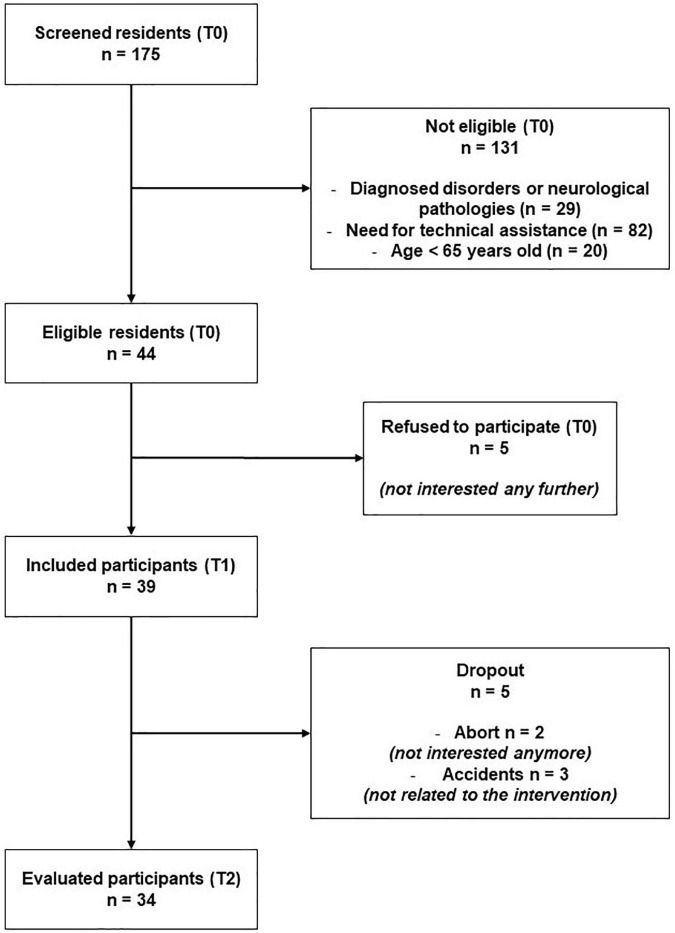
Flowchart of the study.

Among 39 older adults engaged in our study (T1), 34 participants realized the training program until the end as well as final evaluations (T2). This represents an 87% completion rate. Participants’ flow is detailed in Figure 2. Overall, participants realized 23 among 26 mean proposed sessions, representing an 88% attendance rate. Finally, we reported no adverse event throughout the program (286 proposed sessions upon 11 groups, i.e., 8,580 min of training). Thus, the safety level of our exergame was 100%.

### 3.3. Potential effects

Outcomes data and comparison are presented in Table 1. A statistically significant difference between T1 and T2 for several parameters including mental inhibition in single-task condition (Stroop error rate, *p* = 0.007) or postural control in single-task condition (BBS, *p* = 0.014) was found. However, in a desire to improve the robustness of our statistical analysis, we retained only the most significant results.

**TABLE 1:** Difference before (T1) and after (T2) 12 weeks of exergaming in 34 participants.

Outcomes	T1	T2	*d*_T1–T2_	*p*_T1–T2_
**Cognitive**				
**Stroop ST**				
Duration (s)	223 ± 79	220 ± 76	-0.00	0.912^μ^
% of errors	8.0 [5.0; 16.0]	7.0 [2.0; 10.3]	-0.44	**0.007**
TMT (B-A) – duration (s)^§^	145 ± 79	142 ± 67	-0.04	0.439^μ^
1 N-Back – % of errors^§^	56.7 [33.3; 76.7]	20.0 [1.7; 50.0]	1.19	<0.001*
**Motor**				
Postural control in ST (mm.s^–1^)^§^	20.80 [13.67; 25.92]	19.53 [13.67; 30.64]	0.06	0.208
TUG – duration (s)	13.4 [10.3; 17.1]	13.1 [11.5; 18.1]	0.17	0.741
BBS [0; 56]^§^	44.3 ± 8.2	47.4 ± 8.9	0.05	**0.014^μ^**
**Dual task**				
Postural control in DT (mm.s^–1^)^§^	21.43 [14.54; 29.76]	20.73 [15.64; 29.30]	0.23	0.254
Stroop DT – % of errors^§^	15.0 [7.7; 30.1]	9.5 [2.3; 16.5]	-0.54	0.002*
**Characteristics**				
**Quality of life – EQ5D5L**				
Mobility [1; 5]^§^	2.0 [1.0; 3.0]	2.0 [2.0; 3.0]	0.23	0.110
Autonomy [1; 5]^§^	1.0 [1.0; 2.0]	1.0 [1.0; 2.0]	0.08	0.499
Daily activities [1; 5]^§^	1.0 [1.0; 3.0]	1.0 [1.0; 2.0]	-0.03	0.953
Pain [1; 5]^§^	2.5 [2.0; 3.0]	3.0 [2.0; 4.0]	-0.03	0.793
Depression and anxiety [1; 5]^§^	2.0 [1.0; 3.0]	2.0 [1.0; 3.0]	0.03	0.686
Health (/100)^§^	67.39 ± 18.15	66.19 ± 18.29	-0.25	0.278
**Motivation – EMAPS**				
Intrinsic [3; 21]^§^	16.0 [13.0; 18.0]	15.5 [14.0; 18.0]	-0.12	0.574
Extrinsic integrated [3; 21]^§^	12.0 [9.5; 16.5]	14.0 [8.0; 18.0]	-0.10	0.914^μ^
Extrinsic identified [3; 21]^§^	17.0 [14.5; 19.5]	18.0 [16.8; 19.3]	0.00	0.078
Extrinsic introjected [3; 21]^§^	13.0 [11.0; 15.0]	14.0 [11.0; 16.0]	-0.03	0.279^μ^
Extrinsic external [3; 21]^§^	5.0 [3.0; 8.5]	5.0 [3.0; 8.0]	-0.05	0.943^μ^
A motivation [3; 21]^§^	4.0 [3.0; 9.5]	3.5 [3.0; 6.0]	-0.28	0.536
Fear of falling – FES-I [16; 64]^§^	25 [21; 33]	28 [31; 35]	0.12	0.182

BBS, Berg Balance Scale; DT, dual task; ST, single task; TMT, trail making test; TUG, timed up-and-go test; EMAPS, motivation for physical activity for health scale; EQ5D5L, EuroQol five dimensions five levels; FES-I, Falls Efficacy Scale International. ^§^33 participants. Different tests were used to calculate *p*-values; *t*-tests are highlighted with μ; every other value was calculated using Wilcoxon signed-rank tests. Significant *p*-values are highlighted with bold font. Significant *p*-values after Holm–Bonferroni correction are indicated with an*.

Overall, a statistically significant difference regarding working memory under single-task conditions (*p* < 0.001) was observed, and the median error rate during the realization of a visual 1 N-Back went from 57 to 20%. With a Cohen’s *d* value of 1.19, the difference can be considered as strong. A statistically significant difference regarding mental inhibition under the DT condition (*p* = 0.002) was also observed, and the median error rate during the realization of a Stroop test while standing still on a force platform went from 15 to 9.5%. With a Cohen’s *d* value of −0.54, the difference can be considered moderate.

No other parameter emerged as significantly different between T1 and T2. The center of pressure oscillation speed difference between T1 and T2 for 33 participants was not statistically significant (*p* = 0.254). We did not observe any difference after training on mental flexibility or inhibition, mobility, and on postural control under single or DT conditions. We also did not observe any difference after training on the motivation for physical activity, fear of falling, or quality of life levels of the participants.

## 4. Discussion

In this study, the potential use of our exergame as training support and the feasibility of such training were evaluated. The results’ trends on cognitive-motor performance in older adults were also explored. Overall, our exergaming training program seemed feasible; the safety, engagement, attendance, and completion rates seemed high. Our exergame was also potentially effective in improving cognitive functions and in maintaining motor functions, as well as motivation for physical activity, fear of falling, and quality of life levels in older adults.

### 4.1. Attractivity of our exergame

Engagement (89%), attendance (88%), and completion (87%) of our training program were particularly high compared to the literature. For instance, the completion of usual intervention programs ranges from 65 to 86% (Picorelli et al., 2014), but compliance is much lower, ranging from 58 to 77% (Picorelli et al., 2014) or even less than 50% for longer interventions with frailer participants (Lord et al., 2003). In other words, older adults usually do not give up on the training programs they engage in—they rather simply do not perform them. Engagement is more difficult to estimate because studies rarely evaluate the number of participants in relation to the number of eligible people. Our exergame can be considered appealing and enjoyed due to different factors.

First, we applied gamification principles (Gallou-Guyot et al., 2022a), probably increasing the motivation of participants to participate (Sailer et al., 2013). Second, all sessions were supervised, which is known to enhance adherence (Picorelli et al., 2014). Third, the program was collective and took place directly in community dwellings. While home-based or group-based activities get the same short-term compliance and completion rates (Cyarto et al., 2006; Picorelli et al., 2014), home-based activities seem to get more “have try” engagement, while group-based activities get more long-term commitment (Cyarto et al., 2006). The combination of the two maximized our chances of engagement, both short and long term. Fourth, our training was safe, with no adverse event observed during the 286 proposed sessions (i.e., 8,580 min of training), reinforcing the confidence of participants in our exergame and their will to use it. Finally, the possibility of collectively playing and training during the COVID-19 pandemic cannot be underestimated.

### 4.2. Potential effects of our exergame

Our exergame was found potentially effective for the improvement of cognitive functions in older adults, with significant gains in working memory in single-task conditions and in mental inhibition in DT condition. This is in line with literature describing clearer benefits from exergames on cognition than on motor functions (Gallou-Guyot et al., 2020a), explained by a main neuroplastic hypothesis (Monteiro-Junior et al., 2016). For instance, positive effects on the cognitive aspect in DT were observed, not motor. These results are very close to those of Hiyamizu et al. (2012); in this study, 17 older adults realized DT training two times a week for 12 weeks. Following this training, their postural control decreased on average by 15%, while their rate of correct responses to Stroop increased by 26% when assessed in DT condition. This potential cognitive effect of our exergame, in line with the literature (Gallou-Guyot et al., 2020a), can be explained in different ways: important cognitive and low motor loads proposed to participants with more significant cognitive than motor deficits.

First, the cognitive solicitation of our exergame was probably important. We based our DT training program on recommendations for the prescription of physical activity (American College of Sports Medicine and Pescatello, 2014), fall prevention (Sherrington et al., 2017), and DT training and exergaming in older adults (Gallou-Guyot et al., 2020a) using the “moving while thinking” model (Herold et al., 2018). Moreover, our training program was potentially challenging for working memory, because instructions constantly changed not only over the weeks but also over and within sessions. This required the participants to continuously update their knowledge and their reactions to stimuli using mental flexibility and working memory.

At the same time, the participants presented initial cognitive deficits. They had an average Stroop test duration 48% higher than the average (Van der Elst et al., 2006) and a mean TMT duration 30% higher than normal values (Amieva et al., 2009). This is particularly true for working memory, with a rate of correct responses to the visual 1 N-Back test 40% lower than average (Bopp and Verhaeghen, 2020). These deficits are not surprising given the preferential loss of working memory (Wang et al., 2011; Klencklen et al., 2017) among executive functions (Zhou et al., 2011) during normal aging. Moreover, participants were recruited at the end of the COVID-19 pandemic, which is known to have strongly impacted cognitive functions in older adults (De Pue et al., 2021). A hypothesis is that starting from lower, participants had more room for improvement. This makes our exergame potentially relevant as a tool against aging-related cognitive decline.

Second, the motor solicitation of our exergame was maybe too low to induce gains regarding motor functions. The progression through the program was based on succeeding in the current difficulty. The fact that not all participants made it to the end of the incremental program demonstrates that the difficulty was sufficient for the target audience. The lack of motor difficulty would therefore not emerge from the command, but from the participants’ realization. Since we designed our game as an encouragement to physical activity, we valued participation more than good performance. It is possible that the non-correction of exercises from animators and letting participants self-manage their rests did not permit reaching a sufficient level of physical solicitation. Indeed, it has already been described that feedback on the quality of execution of motor tasks constitutes a key element in the training and learning of motor skills (Schmidt and Wrisberg, 2008)—for some authors, it is even the preponderant role of animators (Hodges and Franks, 2002). In a recent study, a giant game board similar in concept to our exergame was used as a support for the practice of physical activity in older adults but supervised by a professional physical activity trainer (Mouton et al., 2017). They found a significant change in the level of physical activity of people as well as their quality of life, discussing the possible impact of the supervisor’s qualification.

It is also possible that increasingly complex DT situations deteriorated motor performance. This can be explained by a strategy of “cognition first, posture second” already experienced in patients with Parkinson’s disease (Bloem et al., 2006). As the difficulty of the exercises increased within and over the sessions, one way for the participants to maintain their cognitive performance was to reduce their motor performance (e.g., only doing half of the movement). This can be considered a trade-off strategy (Plummer et al., 2014). This hypothesis is interesting because the behavior of the participants would then go against the prioritization of the motor task (Schaefer and Schumacher, 2011) often chosen by healthy older adults in a preservation strategy (Bloem et al., 2001). This is particularly relevant facing the higher cognitive than motor deficits of participants compared to normal values: TUG duration 18–30% higher than normal values (Steffen et al., 2002) and means BBS scores 12–17% below standards (Steffen et al., 2002). Knowing that we did not prioritize cognitive tasks during instructions, this chosen strategy from participants can be explained because they felt confident enough to no longer prioritize the motor task.

Finally, the content of the training program may have had qualitative shortcomings with a lack of aerobic component and too much static work [refer to Fraser et al. (2017) for motor effects of aerobic DT training]. This difference between physical training and motor training has already been pointed out by Temprado (2021) in his proposal for defining parameters of effectiveness for exergames.

Nevertheless, our exergame did not lead to gains but potential maintenance regarding motor functions and levels of motivation for physical activity, fear of falling, and quality of life. This can partly be explained by participants’ initial characteristics: motivated, not afraid to fall (Delbaere et al., 2010), and with good quality of life (Van Wilder et al., 2022), therefore difficult to impact. This potential finding would, however, be relevant knowing that the degradation of motor skills such as postural control and mobility is not only a source of discomfort in the daily activities of older adults but also a risk factor for falls leading to limitations in social participation and autonomy loss (Anton et al., 2015). Thus, the maintenance of these capacities can be considered a success in a geriatric context, because many motor parameters such as walking, mobility, and postural control are criteria that define the passage below the threshold of frailty (Sternberg et al., 2011). This is all the more true in the context of the COVID-19 pandemic, which has had a major impact on the activity level and cognitive functions of older adults, among other things (De Pue et al., 2021).

### 4.3. Limits

The first limit of our study is inherent to the pilot study design. The sample size and the lack of a control group do not allow us to bring conclusions about the effects of our exergame but trends. We can only conclude about the feasibility of our specific apparatus, used as training support in a very specific sample, under the controlled conditions of our experiment. However, the feasibility of such intervention at the participants’ living place, supervised by animators (i.e., not physical trainers) constitutes an interest and a solid basis for a future randomized controlled trial.

The second limit of our study was the absence of profiling of our sample depending on cognitive aspects, leading to a very heterogeneous sample and no cutoff value in inclusion criteria. This was reduced by our initial evaluation including many executive functions, as well as the requirement for people to present a score greater than or equal to 5 on the French Gerontologic Autonomy Scale in order to remain within the community dwellings. This grid qualifies the level of autonomy of a person on a scale of 1–6. Level 5 corresponds to total mental autonomy, the person needing only occasional help for the toilet, the preparation of meals, and housekeeping (Coutton, 2001).

The third limit of our study was the difference between the number of training sessions intended and offered (30 vs. 26), due to unpredictable human or material availability. Although this inevitably reduced the training dose by 13%, probably reinforcing the insufficient motor load of our training, this result remains good for an intervention proposed in a difficult health context.

The final limit was inherent to evaluation modalities. First, the tests used were highly sensitive to tests–retests or learning effects. Moreover, most of the exercises performed during our training were dynamic postural control exercises in motion with incorporated cognitive tasks, while our primary outcome was static postural control with an added cognitive task. This choice was justified by the will to compare our results to the literature and to calculate our sample size (Hiyamizu et al., 2012; Fraser et al., 2017). It is possible that we assessed the transfer of training benefits from dynamic to static postural control, rather than actual benefits. This notion of benefits’ transfer has been mentioned by many authors (Gallou-Guyot et al., 2020a). This study could, therefore, support the choice of a more adapted primary outcome later.

### 4.4. Future studies

Facing the potential cognitive impact of our exergame, a randomized controlled trial with a higher number of participants, based on the effect size we measured, should be realized in the future. This study would be an opportunity to compare our exergame to cognitive training known to be effective for cognitive enhancement (Peretz et al., 2011; Kelly et al., 2014), as well as to motor training known to be effective for motor maintenance (Zijlstra et al., 2010; Lesinski et al., 2015).

The technology we used and developed (the “Virtual Carpet”) has already been used to assess visuospatial working memory (Perrochon et al., 2018; Gallou-Guyot et al., 2020b; Kronovsek et al., 2020). In the future, this tool could serve as DT spatial navigation support including various cognitive tasks evaluation (reaction time, working memory, and mental flexibility) for the early diagnosis of cognitive disorders. In parallel, our exergame should also be assessed as training support in other populations in which we found exergaming (Gallou-Guyot et al., 2020c) realized at home (Perrochon et al., 2019; Dalmazane et al., 2021; Gallou-Guyot et al., 2022b) effective for the improvement of varied cognitive and motor outcomes. Our solution was proposed directly in community dwellings and would only require a slight adaptation of the living space at home. The possibility for exergames to be used as home telerehabilitation services is a recent and of interest topic (Meulenberg et al., 2022).

## 5. Conclusion

We conceptualized and developed an exergame that meets the needs of older adults, targeting cognitive and motor risk factors for falls using the concept of CMI. This pilot study consists of a first step; our exergame was found feasible as training support and seemed safe and enjoyed by participants. Our exergame may induce cognitive benefits and help maintain motor functions, motivation for physical activity, fear of falling, and quality of life levels in older adults. These results should be considered with caution regarding the reduced sample size, the lack of a control group, and the highly sensitive measures to test–retests or learning effects we used. However, this is the first step for the specific apparatus used at the living place and supervised by animators (i.e., not physical trainers). All this taken together may represent a particularly suitable and accessible solution that should be further studied, and a new argument for the use of exergame in an active aging perspective.

## Data availability statement

The datasets presented in this article are not readily available because the study was carried amongst an industrial partnership. Requests to access the datasets should be directed to MG-G, matthieu.gallou.guyot@gmail.com.

## Ethics statement

The studies involving human participants were reviewed and approved by Sud-Est 2 (specific reference number: 2020-A02805-34). The patients/participants provided their written informed consent to participate in this study.

## Author contributions

MG-G: conceptualization, methodology, writing – original draft, illustrations, evaluations, animation, and supervision. SM and AP: conceptualization, methodology, writing – corrections, and project management. RM: development and writing – corrections. LR: writing – corrections, evaluations, and animation. J-CD: writing – corrections and project management. All authors contributed to the article and approved the submitted version.
